# *Peony Pollen* Protects against Primary Dysmenorrhea in Mice by Inhibiting Inflammatory Response and Regulating the COX2/PGE2 Pathway

**DOI:** 10.3390/ijms242417245

**Published:** 2023-12-08

**Authors:** Xu Yang, Yunyuan Tian, Jincai Liu, Yaoyao Kou, Yanhua Xie, Siwang Wang, Ye Zhao

**Affiliations:** The College of Life Science, Northwest University, Xi’an 710069, China; 202121749@stumail.nwu.edu.cn (X.Y.); tianyunyuan6861@163.com (Y.T.); kyy211204@163.com (Y.K.); xieyanh@fmmu.edu.cn (Y.X.)

**Keywords:** peony pollen, primary dysmenorrhea, COX-2/PGE2 pathway, inflammatory response, oxidative stress

## Abstract

Peony pollen contains multiple nutrients and components and has been used as a traditional Chinese medicine with a long history, but the effect of the treatment of primary dysmenorrhea remains to be clarified. The aim of this study is to investigate the therapeutic effect of peony pollen on primary dysmenorrhea mice and the potential mechanism. A uterus contraction model in vitro and primary dysmenorrhea mice were used to evaluate the treatment effect of peony pollen on primary dysmenorrhea. The primary dysmenorrhea mice were treated with 62.5 mg/kg, 125 mg/kg, or 250 mg/kg of peony pollen, and the writhing response, latency period, histopathological changes in the uterus, prostaglandin E2 (PGE2) and prostaglandin F2α (PGF2α) levels, and infiltration of neutrophils and macrophages were investigated. Protein expression of interleukin 1 β (IL-1β), interleukin 6 (IL-6), NOD-like receptor thermal protein domain associated protein 3 (NLRP3), cyclooxygenase-2 (COX-2), microsomal prostaglandin-E synthase 1 (mPGEs-1), BCL2-Associated X (Bax), B-cell lymphoma-2 (BCL-2), caspase-3, and cleaved caspase-3 were detected by Western blot, and the oxidative stress related marker malondialdehyde (MDA), superoxide dismutase (SOD), glutathione peroxidase (GSH-Px), and reactive oxygen species (ROS) were evaluated. Peony pollen could attenuate spontaneous or oxytocin-induced uterus contractions in vitro. Moreover, peony pollen decreased the writhing times, prolonged the writhing latency, and reduced the pathological damage of uterine tissues. Furthermore, the inflammatory cell infiltration and the protein expression of IL-1β, IL-6, and NLRP3 were decreased. The COX-2/PGE2 pathway was inhibited; oxidative stress and apoptosis in the uterus also improved in the uterus of primary dysmenorrhea mice. Peony pollen exerts a positive effect on primary dysmenorrhea by inhibiting the inflammatory response and modulating oxidative stress and apoptosis by regulating the COX-2/PGE2 pathway.

## 1. Introduction

Primary dysmenorrhea is the most common gynecological disease in women, defined as menstrual pain without obvious pelvic organic lesions, and usually occurs for the first time within 6–24 months after menarche in women [1]. A survey of female college students showed that the prevalence of primary dysmenorrhea was 85.7% in Saudi Arabia, 85.4% in Ethiopia, 64% in Mexico, 89.1% in Iran, and 41.7% in China [2]. In severe cases, nausea, vomiting, feet, sweating, and even syncope may occur, which affects the quality of female patients’ lives and work. It has been reported that about 50–90% of women suffer from primary dysmenorrhea, and more than 15% are absent from their work, school, and other activities due to severe dysmenorrhea symptoms [3]. At present, the first-line drugs for primary dysmenorrhea are nonsteroidal anti-inflammatory drugs and hormonal therapy, including oral contraceptives or progesterone [4]. However, these medications need to be taken regularly when dysmenorrhea occurs and have common adverse effects, including indigestion, nausea, and headaches [5]. Therefore, it is urgent to explore an alternative medicine with low toxicity to ease symptoms of primary dysmenorrhea.

The pathogenesis of primary dysmenorrhea is not fully understood. The prostaglandins prostaglandin F2α (PGF2α) and prostaglandin E2 (PGE2) play an important role in the development of primary dysmenorrhea [6]. Cyclooxygenase (COX) is involved in the synthesis of prostaglandins [7]. COX-2 activation is the first step in the lipid production of PGE2, which binds to the prostaglandin E receptor2, causing the excessive production of PGF2α and PGF2α, which results in spasmodic contraction of uterine smooth muscle, ischemia, hypoxia, and other pathological changes, leading to pain symptoms [8]. The activation of the COX-2/PGE2 pathway affects the development of primary dysmenorrhea, promotes inflammatory responses, and triggers oxidative stress [9].

Primary dysmenorrhea, of which the pathogenesis is Qi and blood stasis, adopts the treatment of promoting blood circulation and resolving stasis based on basic theories of Chinese medicine [10]. *Moutan Cortex*, the dried root bark of *Paeonia* × *suffruticosa* Andrew, is widely used in gynecological diseases such as primary dysmenorrhea and endometriosis [11]. Peony pollen was collected from the flower of *Paeonia* × *suffruticosa* Andrew and contains similar components such as flavonoids, polyphenols, hormones, and organic acids to the *Moutan Cortex* [12]. It has been recorded that peony pollen also has the effect of promoting blood circulation and eliminating stagnancy and was used as health food and medicine to prevent and treat menstrual pain [13]. However, the therapeutic effect and mechanism of peony pollen on primary dysmenorrhea remains to be elucidated. Therefore, in this study, we evaluated the therapeutic effects of peony pollen in mice with primary dysmenorrhea and illustrated the potential mechanisms.

## 2. Results

### 2.1. Chemical Analysis of Peony Pollen

**Figure 1:**
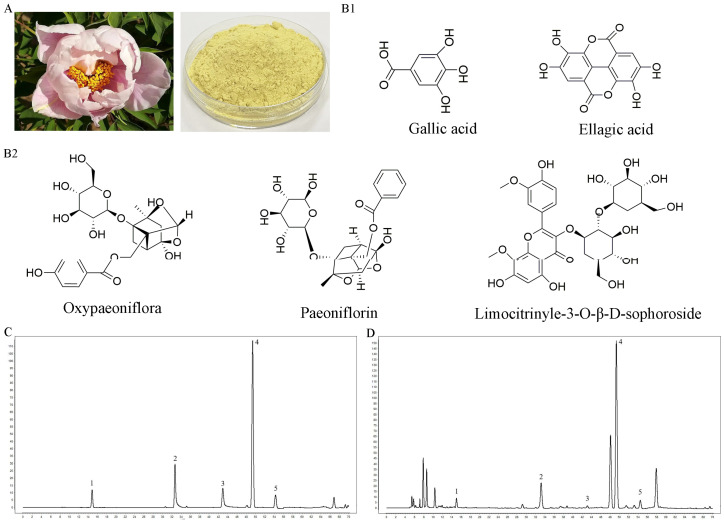
Chemical composition analysis of Peony pollen. (**A**) Peony pollen; (**B1**,**B2**) the chemical structures of five components in peony pollen; (**C**) chromatogram of mixed standard of five components; (**D**) chromatogram of peony pollen analyzed by high-performance liquid chromatography. The number in (C) and (D) is 1. gallic acid, 2. oxypaeoniflora, 3. paeoniflorin, 4. limocitrinyle-3-O-β-D-sophoroside, 5. ellagic acid.

The chemical ingredients of peony pollen powder (PPWE) were detected via high-performance liquid chromatography (HPLC), and the chromatogram is shown in Figure 1. A total of five compounds were identified in the peony pollen, namely gallic acid, oxypaeoniflorin, paeoniflorin, limocitrinyle-3-O-β-D-sophoroside, and ellagic acid (Figure 1). The methodological investigation of HPLC for peony pollen showed that the quantitative method had good linearity, precision, stability, and average recovery (Supplementary Tables S1–S5). Then, the content of five compounds was determined via HPLC and shown in Table 1. The content of gallic acid, oxypaeoniflorin, paeoniflorin, limocitrinyle-3-O-β-D-sophoroside, and ellagic acid in peony pollen was 0.491 mg/g, 3.417 mg/g, 2.443 mg/g, 13.504 mg/g, and 0.562 mg/g, respectively.

### 2.2. Peony Pollen Inhibits Mice Uterine Contraction In Vitro

**Figure 2:**
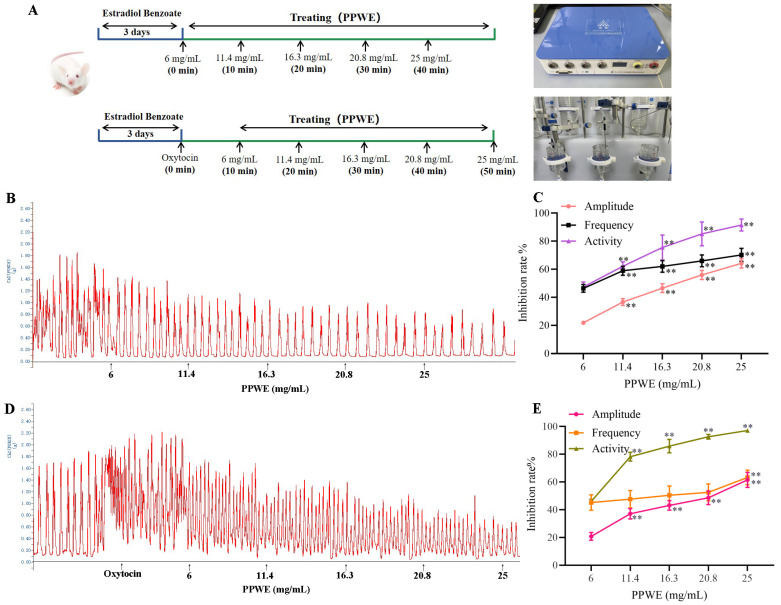
The effect of peony pollen on uterine contraction in vitro. (**A**) The flowchart of the uterus contraction experimental steps; (**B**) representative physiographic recording of uterus spontaneous contractions, with inhibition rate shown in line chart (**C**); (**D**) representative physiographic recording of uterus contractions induced by oxytocin, with inhibition rate shown in line chart (**E**). PPWE: peony pollen powder. ** *p* < 0.01 vs. 6 mg/mL PPWE group (*n* = 6).

Uterine contraction in vitro is widely used for evaluating drug efficacy on dysmenorrhea, and the oxytocin-induced strong and long-lasting uterine contractions are similar to the pathological state of the uterus in patients with dysmenorrhea. The effect of PPWE on spontaneous and oxytocin-induced uterine contractions was evaluated. As shown in Figure 2, PPWE inhibited the spontaneous contractions of isolated uterine smooth muscle in vitro. The contraction amplitude, frequency, and activity decreased after 6–25 mg/mL PPWE treatment in a dose dependent manner. The effective concentration of 50% (EC50) of PPWE on spontaneous contraction was 20.5 mg/mL, and the inhibition rate of 25 mg/mL PPWE on uterine activity reached 80%. After pre-incubation with oxytocin, the uterine contraction was significantly enhanced, and PPWE blocked oxytocin-induced uterine contractions, including amplitude, frequency, and activity. The EC50 of PPWE on oxytocin-induced contraction was 23.1 mg/mL, and the inhibition rates of 25 mg/mL PPWE on uterine activity, contraction amplitude, and frequency were 97.1%, 61.5%, and 63.5%, respectively.

### 2.3. Peony Pollen Ameliorates Pathological Symptoms in Dysmenorrhea Mice

**Figure 3:**
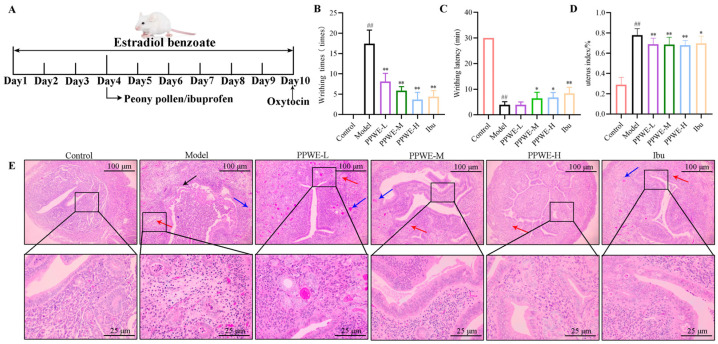
The effect of peony pollen on pathological symptoms of dysmenorrhea mice. (**A**) The flowchart of the animal experiment; (**B**) writhing times of mice after oxytocin injection (*n* = 10); (**C**) writhing latency of mice after oxytocin injection (*n* = 10); (**D**) uterus index (*n* = 10); (**E**) representative images of H&E staining (*n* = 4), → endometrial epithelial damage; → glandular inflammatory exudate; → inflammatory cell infiltration. ## *p* < 0.01 vs. control group; * *p* < 0.05 vs. model group; ** *p* < 0.01 vs. model group. PPWE-L: peony pollen low-dose; PPWE-M: peony pollen low-dose; PPWE-H: peony pollen low-dose; Ibu: ibuprofen.

Writhing times and latency are important indicators for evaluating dysmenorrhea in mice. Compared to the control group, the mice in the model group showed a writhing response about 5 min after the injection of oxytocin, and the number of writhing was about 20 times within 30 min (Figure 3). Furthermore, the uterus index increased, and the endometrial monolayer columnar epithelium and tissue structure were destroyed in the uterus of mice in the model group (Figure 3), indicating that the primary dysmenorrhea model was successfully established. Compared with the model group, the peony pollen middle-dose (PPWE-M)-administered group could reduce the writhing times and prolonged latency in dysmenorrhea mice, and the treatment effect was close to that of the ibuprofen (Ibu) treatment group. As shown in Figure 3, PPWE reduced the uterus index significantly, and the histological lesions of the uterus were alleviated. In the peony pollen low-dose (PPWE-L), PPWE-M, and Ibu groups, there were still glandular inflammatory exudates, but the inflammatory cells were reduced, and the edema was alleviated. The infiltration of inflammatory cells in the uterus of mice in the peony pollen high-dose (PPWE-H) group was reduced, the edema was alleviated, and there was no inflammatory exudate in the glandular cavity.

### 2.4. Peony Pollen Reduced the Inflammatory Response in Dysmenorrhea Mice

**Figure 4:**
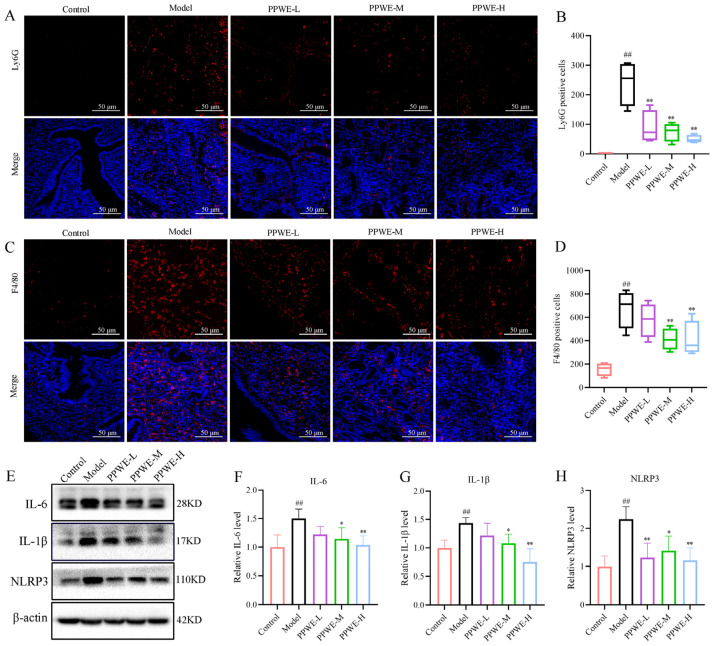
Peony pollen reduces the inflammatory response of dysmenorrhea mice. (**A**) Representative images of neutrophils in the uterus marked by Ly6G, with the bar chart shown in red is for Ly6G, blue is for DAPI (**B**); (**C**) representative images of macrophages in uterus marked by F4/80, with the bar chart shown (red is for F4/80; blue is for DAPI) (**D**); (**E**) the proteins expression of IL-6, IL-1β, and NLRP3 in uterus detected by Western blotting; (**F**–**H**) the gray intensity analysis of IL-6, IL-1β, and NLRP3. ## *p* < 0.01 vs. control group; * *p* < 0.05 vs. model group; ** *p* < 0.01 vs. model group (*n* = 3). PPWE-L: peony pollen low-dose; PPWE-M: peony pollen low-dose; PPWE-H: peony pollen low-dose; Ibu: ibuprofen.

Reduced inflammatory cell infiltration of the uterus by PPWE treatment was observed in hematoxylin and eosin staining (H&E) staining, and then the macrophages and neutrophils were further marked with adhesion G protein-coupled receptor E1 (F4/80) and lymphocyte antigen 6 complex (Ly6G), and the protein expression of inflammatory cytokines was detected in the uterus. As shown in Figure 4, compared with the control group, a large number of macrophages and neutrophils infiltrated the uterus of mice in the model group, and the difference was statistically significant (*p* < 0.01). Compared with the model group, the PPWE-M and PPWE-H administration groups could reduce the macrophages in the uterus of mice (*p* < 0.05 or *p* < 0.01), while PPWE-L had a tendency to reduce the macrophages in the uterus, but was not statistical significance (*p* > 0.05). Furthermore, PPWE-L, PPWE-M, and PPWE-H could reduce neutrophils in the uterus of dysmenorrhea mice (*p* < 0.01). Western blot showed that the inflammatory protein expression also increased in the model group compared with the control group. Interestingly, PPWE could also reduce the inflammatory protein expression of interleukin 1 β (IL-1β), interleukin 6 (IL-6), and NOD-like receptor thermal protein domain associated protein 3 (NLRP3) of the uterus in dysmenorrhea mice in a dose-dependent manner (Figure 4).

### 2.5. Peony Pollen Treatment Inhibiting COX-2/PGE2 Pathway in Dysmenorrhea Mice

**Figure 5:**
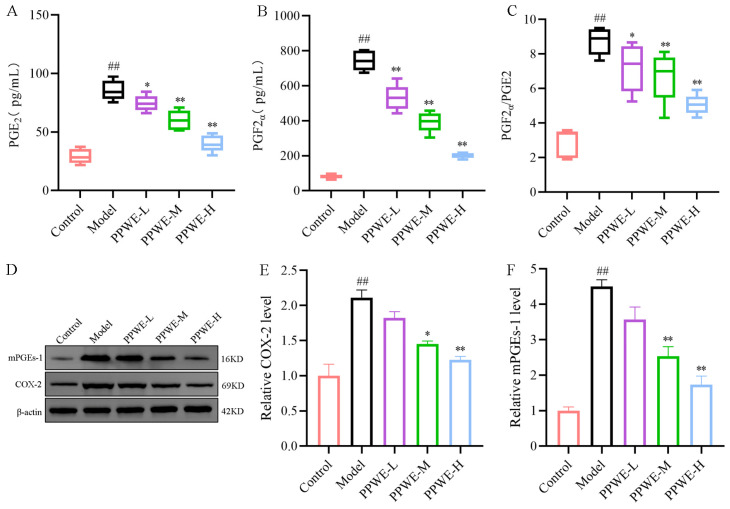
Peony pollen treatment inhibiting COX-2/PGE2 pathway in dysmenorrhea mice. (**A**) PGE2 level in uterus (*n* = 6); (**B**) PGF2α levels in uterus (*n* = 6); (**C**) the ratio of PGF2α/PGE2 (*n* = 6); (**D**) the proteins expression of COX-2 and mPGEs-1 in uterus detected by Western blotting; (**E**,**F**) the gray intensity analysis of COX-2 and mPGEs-1. ## *p* < 0.01 vs. control group; * *p* < 0.05 vs. model group; ** *p* < 0.01 vs. model group (*n* = 3). PPWE-L: peony pollen low-dose; PPWE-M: peony pollen low-dose; PPWE-H: peony pollen low-dose; Ibu: ibuprofen; PGE2: prostaglandin E2; PGF2α: prostaglandin F2α; COX-2: cyclooxygenase-2; mPGEs-1: microsomal prostaglandin-E synthase 1.

COX-2 is involved in inflammation and pain response by catalyzing the production of PGE2 from arachidonic acid. The PGE2 and PGF2α levels in the uterus were detected using the enzyme-linked immunosorbent assay (ELISA) kit. As shown in Figure 5, compared with the control group, the PGE2 and PGF2α content of the uterus increased, and the ratio of PGF2α/PGE2 was also elevated in the model group. Interestingly, PPWE-L, PPWE-M, and PPWE-H decreased the PGE2, PGF2α levels, and ratio of PGF2α/PGE2 in the uterus. The protein expression of COX-2 and microsomal prostaglandin-E synthase 1 (mPGEs-1) was further detected by Western blot. As shown in Figure 5, the protein expression of the COX-2 and mPGEs-1 in the uterus of dysmenorrhea mice increased compared with the control group, which indicated that the COX-2/PGE2 pathway was activated in dysmenorrhea mice. Compared with the model group, PPWE treatment was also able to downregulate the protein expression of COX-2 and mPGEs-1 in a dose-dependent manner, thereby inhibiting the activation of the COX-2/PGE2 pathway.

### 2.6. Peony Pollen Alleviates Oxidative Stress in Dysmenorrhea Mice

**Figure 6:**
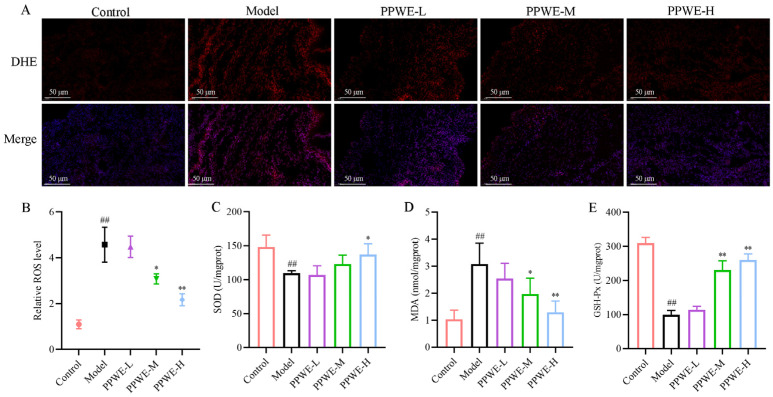
Peony pollen alleviates oxidative stress in dysmenorrhea mice. (**A**) Dihydroethidium (DHE) staining of the uterus for detection of ROS, with the bar chart of ROS level shown in (*n* = 3) (**B**). Red is for ROS; the merge is for ROS (red) and DAPI (blue); (**C**) SOD activity of uterus (*n* = 6); (**D**) MDA level in uterus (*n* = 6); (**E**) GSH-Px activity of uterus (*n* = 6). ## *p* < 0.01 vs. control group; * *p* < 0.05 vs. model group; ** *p* < 0.01 vs. model group. PPWE-L: peony pollen low-dose; PPWE-M: peony pollen low-dose; PPWE-H: peony pollen low-dose; Ibu: ibuprofen; ROS: reactive oxygen species; SOD: superoxide dismutase; MDA: malondialdehyde; GSH-Px: glu-tathione peroxidase.

COX-2 is an important oxidative stress-associated factor that causes the uterus to be in a state of oxidative stress in dysmenorrheal patients by promoting the overproduction of PGE2. We further analyzed the anti-oxidative stress effect of PPWE in dysmenorrhea mice. Compared to the control group, the reactive oxygen species (ROS) level in the model group was raised, and PPWE treatment dramatically inhibited ROS levels in the uterus of dysmenorrhea mice, especially the PPWE-H treatment group (Figure 6). Superoxide dismutase (SOD), oxidative stress-related marker malondialdehyde (MDA), and glutathione peroxidase (GSH-Px) content were also detected in the uterus, which was consistent with the results of ROS staining. Compared with the control group, the SOD, MDA, and GSH-Px increased significantly in the model group and were effectively decreased after the administration of different doses of PPWE compared with the model group (*p* < 0.05 or *p* < 0.01), which indicates that PPWE alleviates dysmenorrhea by reducing uterine oxidative stress.

### 2.7. Peony Pollen Alleviates Endometrial Epithelial Cell Apoptosis in Dysmenorrhea Mice

**Figure 7:**
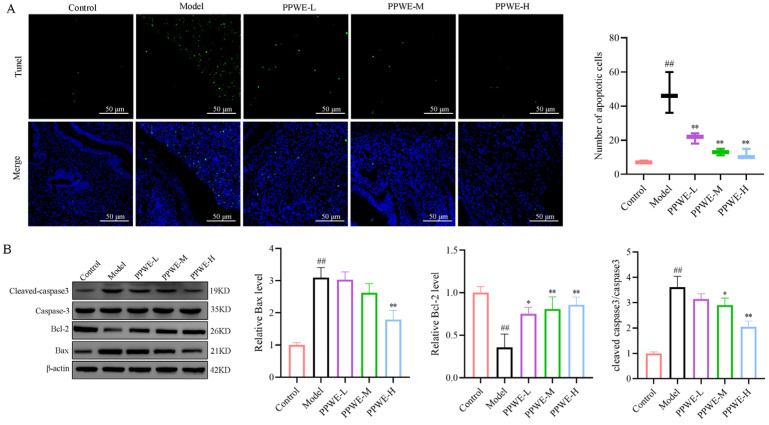
Peony pollen alleviates endometrial epithelial cell apoptosis in dysmenorrhea mice. (**A**) Tunel staining of the uterus for detection of apoptotic cells, green is for Tunel, and blue is for DAPI (*n* = 3). (**B**) The protein expression of Bax, Bcl-2, caspase-3, and cleaved-caspase-3 in uterus detected by Western blotting. ## *p* < 0.01 vs. control group; * *p* < 0.05 vs. model group; ** *p* < 0.01 vs. model group (*n* = 3). PPWE-L: peony pollen low-dose; PPWE-M: peony pollen low-dose; PPWE-H: peony pollen low-dose; Ibu: ibuprofen.

Activation of the COX-2/PGE2 pathway induces oxidative stress inflammatory factor releases, leading to the development of apoptosis. Tunel staining shows that compared with the control group, the model group had more apoptotic cells in the uterus and uterine cavity, and PPWE treatment reduced apoptotic cells in the uterus of dysmenorrhea mice compared with the model group (Figure 7). Western blotting analysis further verified the role of PPWE in regulating apoptosis. As shown in Figure 7, compared with the control group, the pro-apoptotic protein expression of BCL2-Associated X (Bax) and cleaved-caspase-3 were upregulated, while the anti-apoptotic proteins of Bcl-2 were downregulated in the uterus of the model group (*p* < 0.01). After being administrated with different concentrations of PPWE, the protein expression of Bax and cleaved-caspase-3 was lower than the model group, and the B-cell lymphoma-2 (BCL-2) protein expression increased (*p* < 0.05 or *p* < 0.01). This result suggested that PPWE alleviated the endometrial epithelial cell apoptosis by modulating protein expression.

## 3. Discussion

As primary dysmenorrhea is pain without any organic cause and is not life-threatening, women with primary dysmenorrhea have received less scientific and clinical attention [14]. Women with dysmenorrhea are more likely to experience emotions such as depression, anxiety, and negative self-perception than women without dysmenorrhea [15]. Although Western medicines are quick and effective ways of relieving pain, they need to be taken repeatedly with the menstrual cycle, and it is difficult to improve the patient’s fear and anxiety caused by recurrent menstrual pain. The “concept of holism” and “treatment of diseases must concentrate on the root cause” of traditional Chinese medicine have distinct advantages in the treatment of primary dysmenorrhea. The “gynecological instructions” complied into the Qing dynasty, records “premenstrual abdominal pain, the stagnation of qi and blood, postmenstrual stabbing pain, blood room of the virtual” and has been used to the present day [16].

The peony is China’s national flower; its pollen, like the other medicinal parts of the peony, has the effect of promoting blood circulation to regulate menstruation. It has been used as health food and medicine to prevent and treat menstrual pain since ancient times because of redundant nutrient ingredients and the character of the homology of medicine and food [17]. However, the therapeutic effect and mechanism of peony pollen on primary dysmenorrhea remain uncovered, which hinders the development of modern medicine for the treatment of primary dysmenorrhea. A uterus contraction model in vitro and a primary dysmenorrhea mice model in vivo were employed in this study, and the efficacy of PPWE on primary dysmenorrhea was revealed. We found that PPWE inhibited the uterus’s spontaneous or oxytocin-induced contraction in vitro, decreased the writhing times, prolonged the latency period, and improved the pathological damage of the uterus in primary dysmenorrhea mice.

Menstruation was regarded as an inflammatory response because of the leukocytic and inflammatory mediator production during menstruation [18]. Metalloproteinases (MMPs) produced by endometrial cells and leukocytes are important enzymes regulate the exfoliation of endometrium [19]. And the infiltration of leukocytes also produced pro-inflammatory cytokines such as IL-1β, IL-6, tumor necrosis factor α (TNF-α), and the chemokine of IL-8 in endometrium local to cause endometrial edema and red blood [20]. However, the relationship between primary dysmenorrhea and over-inflammatory responses has raised attention in recent years. The complex pathophysiology induced the activation of nuclear factor kappa-B (NF-κB) to release excessive pro-inflammatory mediators and eventually induce uterus destruction [21]. Therefore, the inflammatory response in primary dysmenorrhea mice was evaluated in this study. The result revealed that PPWE inhibited the excessive infiltration of neutrophils and macrophages in the endometrial layer and decreased the expression of pro-inflammatory protein IL-1β, IL-6, and NLRP3 in the uterus of primary dysmenorrhea mice. This result indicated that PPWE improves primary dysmenorrhea and may be related to mitigating the inflammation.

PGF2α and PGE2 have a vital role in the inflammatory response and are deemed as a basic pathogenesis mechanism for primary dysmenorrhea formation. Cyclooxygenases (COX-1 and COX-2) are rate-limiting enzymes in modulating the synthesis of various endogenous prostaglandins (PGs) from arachidonic acid. Arachidonic acid was catalyzed by COX-2 and mPGEs-1 to the production and biosynthesis of the PGE2 in inflammation or was converted to prostaglandin H2 (PGH2) for PGF2α generation [22]. PGF2α contributes to the abnormal spasmodic contraction of uterine smooth muscle by vasoconstriction to reduce blood flow, ultimately causing ischemia and pain during menstruation. PGE2 mediated by PGE2 receptor2 may even aggravate uterus edema and leukocyte recruiting. Higher rates of PGF2α/PGE2 levels were reported in primary dysmenorrhea patients in clinical studies [23]. COX-2/PGE2 pathway activation and increasing levels of PGF2α, PGE2, and PGF2α/PGE2 were observed in primary dysmenorrhea mice, and PPWE decreased the level of PGE2 and PGF2α in the uterus by inhibiting the expression of COX-2 and mPGEs-1 proteins, which are the major regulatory protein in COX-2/PGE2 pathway.

Oxidative stress is closely related to the pathogenesis of multiple diseases. The levels of the most oxidative stress markers were abnormally high in primary dysmenorrhea women [24]. Oxidative stress markers, such as lipid peroxidation products MDA and reactive nitrogen species NO, were elevated in primary dysmenorrhea patients compared to healthy women, along with a decreased activity of GSH-Px [25,26]. Previous research indicated that PGE2 exerts immunosuppressive effects in macrophages, and PGE2 pathway activation modulated the oxidative stress-induced ferroptosis in epithelial cells [27]. Moreover, PGE2 was significantly elevated in primary dysmenorrhea patients, and our established model mice, so the oxidative stress levels in mice were evaluated. The results revealed that oxidative stress of primary dysmenorrhea mice with elevated ROS and MDA levels, and decreased activity of SOD and GSH-Px enzyme, whereas PPWE reduced the oxidative stress in primary dysmenorrhea mice by reducing ROS and MDA production, increasing SOD and GSH-Px activity.

Menstruation is a delicate balance of many physiological processes, such as proliferation, inflammation, hypoxia, vasoconstriction, and apoptosis [28]. Although patients with primary dysmenorrhea do not present with organic pathology, this delicate balance is disrupted by excessive inflammatory cell infiltration, the release of inflammatory factors such as IL-1β, IL-6, and NLRP3, over-expression of COX-2, increased levels of PGE2, and subsequent oxidative stress of organisms could also alleviate the apoptosis level [29]. Once the delicate balance of multiple physiological processes is disrupted, it may cause or exacerbate dysmenorrhea symptoms. Our data uncovered that PPWE could alleviate the unbalance of apoptosis in primary dysmenorrhea mice by modulating apoptosis protein expression.

In conclusion, peony pollen alleviated the symptoms of primary dysmenorrhea mice, and the mechanism is related to decreasing the inflammatory cell infiltration and the production of pro-inflammatory cytokines, regulating the COX-2/PGE2 pathway to reduce oxidative stress and apoptosis.

## 4. Materials and Methods

### 4.1. Preparation of Peony Pollen Mice

The peony pollen was harvested in late April from the peony growing base in Heyang, Shaanxi, China, and was identified by associate chief pharmacist Yanhua Xie. After cleaning and breaking up through an 80-mesh sieve, the peony pollen was then crushed again at a low temperature to make a broken-down wall powder.

### 4.2. Peony Pollen Alleviates Endometrial Epithelial Cell Apoptosis in Dysmenorrhea Mice

#### 4.2.1. Chemical and Reagents

Gallic acid, ellagic acid, oxypaeoniflora, and paeoniflorin were purchased from Baoji Herbest Bio-Technology Company (Baoji, China, purity ≥ 98%). Limocitrinyle-3-O-β-D-sophoroside was prepared in the laboratory via extraction, isolation, and purification. Estradiol benzoate injection was purchased from Sichuan Jinke pharmaceutical company (Chengdu, China, lot: 20220503). Oxytocin injection was purchased from Shanghai Harvest pharmaceutical company (Shanghai, China, lot: 09221204). Mouse prostaglandin F2α (PGF2α, lot: ER0186ZJ4438) and prostaglandin E2 (PGE2, lot: ER004PN81779) ELISA kits were purchased from Elabscience Biotechnology (Wuhan, China). Anti-F4/80 (GB113373), anti-Ly6G (GB1129), anti-caspase-3, anti-Bax, anti-Bcl-2, and anti-β-actin were purchased from Servicebio (Wuhan, China). Anti-mPGEs-1 (ab168621) and anti-COX-2 (ab179800) were purchased from Abcam (Cambridge, MA, USA). Anti-cleaved caspase-3 was purchased from Cell Signaling Technology (9664; Boston, MA, USA). Anti-IL-6 (WL02841), anti-IL-1β (WL00891), and anti-NLRP3 (WL02635) were purchased from Wanleibio (Shenyang, China).

#### 4.2.2. Analysis of Peony Pollen by High-Performance Liquid Chromatographic

A total of 0.50134 g peony pollen powder was dissolved in 50 mL ultrapure water and extracted with ultrasonic for 60 min (250 W, 40 KHz). The supernatant was filtered and analyzed using the Shim-pack GIST C_18_ column (250 mm × 4.6 mm, 5 μm). The mobile phase was ultrapure water with 0.085% phosphoric acid (A) and acetonitrile (B), and the gradient elution program was employed as follows: 0–25 min, 4–14% B; 25–26 min, 14–16% B; 26–46 min, 16–20% B; 46–56 min, 20–40% B; 56–70 min, 40–50% B. The column temperature was 35 °C, the flow rate was 0.6 mL/min, and the injection volume was 10 μL. The spectra were recorded at 270 nm in 0–56 min and changed to 240 nm in 56–70 min. The components of peony pollen were quantified using an external standard method. Methodological analyses such as linear regression for the calibration curve, precision, stability, and average recovery were shown in Supplementary Materials.

#### 4.2.3. Uterus Contraction Test In Vitro

The flowchart of the uterus contraction test steps is shown in Figure 2A. Female BALB/c mice were injected intraperitoneally with 10 mg/kg estradiol benzoate for three consecutive days, and the uterus was removed on the fourth day and washed in Locke’s solution. The uterus in vitro was tied at both ends, and the cervix of the uterus was fixed at the bottom of the bath in the HV1403 Isolated Tissue and Organ Thermostatic Perfusion System (Chengdu Taimeng Software Company, Chengdu, China) and was placed in a thermostatic bath containing 20 mL Locke’s solution. The temperature was maintained at (37 ± 0.1) °C, and a gas mixture of 95% oxygen (O_2_) +5% carbon dioxide (CO_2_). After the contraction stabilized in the uterus, either the normal contraction curve for 10 min was recorded or 5 IU/L oxytocin injection was added and the uterine contraction curve was recorded for 15 min. Then, the cumulative administration method was used by adding peony pollen liquid to the test tubes to make the concentrations of 6, 11.4, 16.3, 20.8, and 25 mg/mL, and the action time of each concentration was 10 min, respectively. The amplitude, frequency, and uterine activity of uterine contraction at different concentrations were recorded using BL-420N, the BL-420N Biofunctional Experiment System (Chengdu Taimeng Software Company, China), calculating the uterine contraction inhibition rate.

#### 4.2.4. Animal Treatment

The flowchart of the animal experiment steps is shown in Figure 3. Eighty specific pathogen-free BALB/c female mice weighing 20–24 g were obtained from the Air Force Medical University Experimental Animal Center (Xi’an, China). All animal experimental procedures were approved by the Laboratory Animal Welfare and Ethics Committee of Air Force Medical University (No. 20191206). Mice were randomly divided into eight groups with 10 mice, including control, model, PPWE-L, PPWE-M, PPWE-H, and Ibu groups. Mice in the control group were injected subcutaneously with olive oil, and mice in the other seven groups were injected with 10 mg/kg estradiol benzoate for ten days [30]. On the fourth day of estradiol benzoate injection, mice in the PPWE-L, PPWE-M and PPWE-H groups were intragastrically administered with peony pollen at 62.5, 125, and 250 mg/kg, respectively, and the positive group was given 121 mg/kg ibuprofen. The dosage of ibuprofen was calculated based on the human clinical dosage (mouse dosage = human daily dosage/60 kg body weight × 9.1).

#### 4.2.5. Behavioral Analysis

On day 10, dysmenorrhea was induced via intraperitoneal injection of 200 IU/kg oxytocin. The mice were placed in transparent and empty cage boxes, the amount of writhing was measured within 30 min, and the time of the first twist was recorded as the latency period. The criteria for writhing response were based on that which was previously described in detail [31].

#### 4.2.6. Detection of PGE2 and PGF2α in Uterus

The uterus was precisely weighed 30 mg before nine times the amount of pre-cooled phosphate-buffered saline (PBS) was added. The tissue homogenate was centrifuged at 4 °C, 5000 r/min for 15 min, and separated the supernatant for subsequent detection. The PGF2α and PGE2 ELISA kit was used to detect the content of PGE2 and PGF2α in the uterus of mice in accordance with the applicable recommendations, respectively.

#### 4.2.7. Detection of SOD, MDA, and GSH-PX in Uterus

The SOD, MDA, and GSH-PX assay standards were diluted into a corresponding concentration gradient using standard diluents, and the appropriate amount was added to the control tube. The supernatant of mice uterus prepared in “4.2.6 detection of PGE2 and PGF2α in uterus” was added into the assay tube. Then, SOD, MDA, or GSH-PX working solution was added, and the absorbance was detected by an enzyme meter after the reaction was finished, according to the manufacturer’s instructions.

#### 4.2.8. Histopathology Analysis

H&E was used to evaluate the histopathological changes in the uterus. The uterus tissue was fixed in paraformaldehyde, dehydrated, and embedded in a paraffin block; after the section was cut into 3–5 µm slices, H&E staining was performed and then observed under a microscope at 100× and 400× magnifications.

#### 4.2.9. Immunofluorescence Analysis

After antigen retrieval, paraffin sections were blocked in bovine serum albumin for 30 min and incubated overnight at 4 °C with primary antibodies of anti-F4/80 and anti-Ly6G. After washing slides three times with phosphate-buffered saline, slides were incubated with 100 μL CY3-labeled secondary antibody (GB21303; Servicebio) in the humidifying box for 60 min. Then, 100 μL of DAPI staining solution was added to stain the nuclei. The fluorescence intensities were measured at λ_ex_/λ_em_ = 510 nm/590 nm with a Nikon Eclipse Ti confocal microscope (Nikon, Tokyo, Japan).

#### 4.2.10. Tunel Analysis

Proteinase K working solution was added to the paraffin slides of the uterus and incubated at 37 °C for 30 min, followed by permeabilization and equilibrium at room temperature. An amount of 200 μL of the mixture (terminal deoxynucleotidyl transferase enzyme, dUTP, and buffer mixed at a 1:5:50 ratio) was taken to cover the uterus tissue and incubated at 37 °C for 2 h. The nucleus was stained with DAPI. The excitation was measured at 465–495 nm, and the emission was measured at 515–555 nm with a fluorescence microscope (Nikon).

#### 4.2.11. Western Blot Analysis

Proteins from the uterus of mice were separated by sodium dodecyl sulfate-polyacrylamide gel electrophoresis and electro-transferred to polyvinylidene fluoride membranes. Then, membranes were blocked in 5% non-fat milk and incubated overnight at 4 °C with the primary antibodies of anti-Il-6, anti-IL-1β, anti-NLRP3, anti-COX-2, anti-mPGEs-1, anti-caspase-3, anti-cleaved caspase-3, anti-Bax, anti-Bcl-2, and anti-β-actin served as the internal control. Then, membranes were incubated with the appropriate secondary antibody for 1 h at room temperature. The intensity of each band was scanned by the ChemiDoc™XRS+ Imaging System and analyzed with ImageJ software 1.54f.

#### 4.2.12. Statistical Analyses

Data were analyzed using IBM’s SPSS 23.0 software. One-way analysis of variance (ANOVA) was performed, followed by post hoc comparisons with the least significant difference (LSD) test between different groups. The data were presented as the mean ± standard deviation, and *p* < 0.05 was considered significantly different.

## 5. Conclusions

To summarize, peony pollen improves the pathological symptoms of primary dysmenorrhea, and the mechanism is related to inhibiting the inflammatory response and modulating oxidative stress and apoptosis by regulating the COX-2/PGE2 pathway.

## Figures and Tables

**Table 1:** The content of chemical components of peony pollen powder (*n* = 4).

Compounds	Content (mg/g)				Mean
	20220912	20220914	20220915	20220917	
Gallic Acid	0.492	0.491	0.494	0.490	0.492
Oxypaeoniflorin	3.442	3.434	3.411	3.383	3.416
Paeoniflorin	2.448	2.472	2.428	2.418	2.442
Limocitrin-3-O-sophoroside	13.462	13.569	13.524	13.128	13.421
Ellagic Acid	0.572	0.566	0.566	0.546	0.563

## Data Availability

The authors confirm that the data supporting the findings of this study are available within the article.

## Supplementary Materials

The following supporting information can be downloaded at https://www.mdpi.com/article/10.3390/ijms242417245/s1.
