# Supplementary material for: Peony Pollen Protects against Primary Dysmenorrhea in Mice by Inhibiting Inflammatory Response and Regulating the COX2/PGE2 Pathway
Source: Int J Mol Sci. 2023 Dec 8;24(24):17245. doi: 10.3390/ijms242417245 (PMC10743473; doi:10.3390/ijms242417245)
Supplement: Supplementary file 1 [file ijms-24-17245-s001.zip › ijms-2718502-supplementary.pdf]

Supplementary Material

Peony pollen protect against primary dysmenorrhea in mice by inhibiting inflammatory response and regulating COX2/PGE2 pathway

Xu Yang ‡, Yunyuan Tian ‡, Jincai Liu, Yaoyao Kou, Yanhua Xie, Siwang Wang * and Ye Zhao *

The College of Life Science, Northwest University, Xi'an 710069, China
‡ Contributed equally to this work
* Correspondence: Siwang Wang, Email address: 20182029@nwu.edu.cn;
Ye Zhao, Email address: zhaoye@nwu.edu.cn

Supplementary Table S1. Regression equations, correlation coefficients, linear ranges, LODs and LOQs, of five components.

Analyte	Regression equation	Correlation coefficient	Linear range (μg /mL)	LODs (μg/ mL)	LOQs (μg/ mL)
Gallic Acid	y = 25.88 x - 3.72	0.9995	0.96-95.84	0.32	0.96
Oxypaeoniflorin	y = 17.34 x + 0.55	0.9992	1.36-135.93	0.73	1.36
Paeoniflorin	y = 1.84 x + 0.32	0.9994	2.47-246.8	1.91	2.47
Limocitrin-3-O-sophoroside	y = 24.73 x + 5.07	0.9994	2.7-269.7	0.62	2.7
Ellagic Acid	y = 30.33 x + 1.14	0.9998	0.57-57.04	0.25	0.57

Supplementary Table S2. Precision of five components (*n* = 6).

Analyte	1	2	3	4	5	6	RSD%
Gallic Acid	121.81	124.91	123.98	121.93	124.27	123.82	1.04
Oxypaeoniflorin	600.81	607.16	609.04	613.30	615.96	602.67	0.97
Paeoniflorin	44.76	43.96	44.15	44.67	44.82	43.99	0.90
Limocitrin-3-O-sophoroside	3486.55	3456.37	3448.99	3446.29	3437.99	3465.52	0.50
Ellagic Acid	177.85	175.97	176.05	176.20	176.72	177.56	0.46

Supplementary Table S3. Repeatability of five components (*n* = 6).

Analyte	1	2	3	4	5	6	RSD%
Gallic Acid	0.490	0.490	0.491	0.491	0.496	0.506	1.24
Oxypaeoniflorin	3.437	3.437	3.408	3.381	3.388	3.429	0.72
Paeoniflorin	2.439	2.456	2.373	2.401	2.440	2.454	1.37
Limocitrin-3-O-sophoroside	13.436	13.568	13.514	13.127	13.595	13.544	1.29
Ellagic Acid	0.569	0.567	0.568	0.550	0.575	0.574	1.61

Supplementary Table S4. Stability of five components (*n* = 6).

Analyte	0h	2h	4h	6h	8h	10h	12h	24h	48h	RSD%
Gallic Acid	142.37	143.80	140.59	142.65	142.43	142.39	142.51	141.97	138.31	1.11
Oxypaeoniflorin	587.46	585.87	586.81	582.66	577.54	577.58	577.00	577.96	579.75	0.75
Paeoniflorin	44.09	43.65	43.47	43.33	43.02	42.78	42.94	42.89	42.35	1.20
Limocitrin-3-O-sophoroside	3381.29	3371.30	3366.46	3361.19	3367.12	3367.01	3356.91	3352.28	3306.46	0.64
Ellagic Acid	175.53	173.32	174.80	171.88	172.61	172.36	172.12	171.82	171.00	0.85

Supplementary Table S5. Recovery of five components (*n* = 6).

Analyte	1	2	3	4	5	6	Mean	RSD%
Gallic Acid	97.79	95.75	96.04	97.6	98	99.7	97.48	1.479
Oxypaeoniflorin	99.78	98.07	97.92	97.62	96.71	97.12	97.87	1.087
Paeoniflorin	99.18	95.33	97.39	96.89	98.66	97.33	97.46	1.398
Limocitrin-3-O-sophoroside	99.61	99.45	97.59	98.24	98.53	96.26	98.28	1.269
Ellagic Acid	98.96	99.22	99.49	95.23	99.51	99.27	98.62	1.692
